# Advancing proton therapy: Dosimetric superiority of proton arc therapy over IMPT in esophageal cancer treatment

**DOI:** 10.1002/acm2.70607

**Published:** 2026-05-05

**Authors:** Xiaoda Cong, Ebin Sebastian, Peilin Liu, Xiaoqiang Li, James Dolan, Raymond Dalfson, Martin Soukup, Etienne Lessard, Rohan L. Deraniyagala, Craig W. Stevens, Peter Y. Chen, Xuanfeng Ding

**Affiliations:** ^1^ Corewell Health William Beaumont University Hospital Royal oak Michigan USA; ^2^ Elekta AB Stockholm Sweden

**Keywords:** esophagus cancer, monaco, proton arc therapy

## Abstract

**Background and aims:**

Esophageal cancer presents significant treatment challenges due to its proximity to critical structures such as the heart, lungs, and spinal cord. While intensity‐modulated proton therapy (IMPT) improves dose conformity, it is limited by factors such as the number of beam angles and lateral penumbra. Proton arc therapy (PAT) may overcome these issues by utilizing continuous gantry rotation to enhance conformity and spare organs‐at‐risk (OARs). This study compared PAT and IMPT in esophageal cancer, focusing on dosimetric outcomes, and dose‐averaged linear energy transfer (LET_d_) distributions.

**Methods:**

A retrospective analysis was conducted on ten esophageal cancer patients with variable tumor characteristics. Treatment plans were created using Monaco v6.1 treatment planning system for both PAT and IMPT, maintaining identical robustness parameters (± 5 mm setup and ± 3.5% range uncertainties). Both modalities were prescribed a total dose of 50.4 Gy(RBE) in 28 fractions, ensuring ≥95% clinical target volume (CTV) coverage in the worst‐case scenarios. Key metrics, including conformity index (CI), heterogeneity index (HI), and LET_d_, were compared. Robustness evaluations were performed across 21 worst‐case scenarios to assess plan quality and OAR sparing.

**Results:**

PAT demonstrated superior dose conformity (CI: 0.67 ± 0.17 vs. 0.54 ± 0.13; *p* < 0.01) and reduced lung V20 (6.88% vs. 13.44%; *p* < 0.01) compared to IMPT. Critical structure sparing, including reduced spinal cord doses (max dose of (30.93 ± 10.85) Gy(RBE) vs (23.22 ± 9.55) Gy(RBE), *p* = 0.02), was achieved without compromising CTV coverage. PAT showed higher LET_d_ within the CTV and lower LET_d_ in adjacent organs‐at‐risk relative to IMPT, without statistically significant differences. While the clinical significance remains uncertain, this pattern may support more refined biological dose shaping.

**Conclusions:**

PAT emerged as a promising modality for esophageal cancer treatment, delivering improved dose conformity and reduced OAR exposure. These advantages suggest PAT's potential to decrease radiation‐associated complications and improve therapeutic outcomes, warranting further clinical validation.

## 1 INTRODUCTION

Esophageal cancer is a significant global health concern, with high rates of morbidity and mortality.^1^ Despite advancements in radiation therapy, treating esophageal cancer remains challenging due to its anatomical proximity to critical structures such as the heart, lungs, and spinal cord.^2^, ^3^ As a result, conventional photon radiation therapy often faces limitations in delivering the necessary dose to the tumor while sparing nearby healthy tissues. The advent of proton therapy has offered a promising alternative, given its ability to provide better dose conformity and reduce radiation exposure to adjacent organs‐at‐risk (OARs) through the exploitation of the Bragg peak effect.^4^, ^5^, ^6^ In addition to dosimetric advantages, emerging clinical evidence suggests that proton therapy may be associated with improved survival outcomes and reduced treatment‐related toxicities in patients with esophageal cancer.^7^

Proton therapy, particularly intensity‐modulated proton therapy (IMPT), has become an established treatment modality for selected cancers, including esophageal cancer supported by growing evidence of dosimetric and clinical advantages over photon‐based techniques.^8^ IMPT offers enhanced precision by modulating proton beam intensities to conform to the tumor's shape.^9^, ^10^ However, despite its advantages, IMPT is not without limitations. Treatment uncertainties related to proton beam range and setup errors, as well as the increased lateral penumbra, pose challenges in maximizing dose conformity while protecting OARs.^11^, ^12^

Recent studies have highlighted that while proton therapy offers improved dose conformity compared to photon‐based techniques, it is not immune to treatment‐related toxicities. For example, pneumonitis has been reported in over 10% of patients undergoing proton therapy for thoracic cancers, particularly when large lung volumes are irradiated.^13^, ^14^ Additionally, cardiac and pulmonary toxicities remain important concerns in thoracic radiotherapy due to incidental irradiation of critical cardiopulmonary structures. In esophageal cancer, this risk is particularly relevant given the close anatomical proximity of the heart and lungs to the target volume.^15^ Importantly, comparative clinical evidence suggests that proton therapy, relative to photon‐based radiotherapy, is associated with reduced cardiopulmonary toxicities, including radiation pneumonitis and pericardial effusion.^7^ Despite these advantages, further improvements in dose shaping and normal tissue sparing remain desirable, motivating investigation of advanced delivery techniques such as proton arc therapy (PAT).

Recent technological advancements have led to the development of PAT, a novel approach that aims to overcome some of these limitations by utilizing continuous gantry rotation to deliver protons from numerous angles.^16^ This technique has demonstrated potential dosimetric benefits, particularly in improving target dose conformity and reducing radiation exposure to OARs in cancers such as prostate, lung, breast, head‐and‐neck, and spine.^17^, ^18^, ^19^, ^20^, ^21^ While recent planning studies have explored PAT for esophageal cancer and demonstrated potential dosimetric advantages, additional validation across planning systems and clinical scenarios remains necessary.^22^

In this study, we aimed to investigate the potential clinical benefits of PAT for esophageal cancer, focusing on its ability to spare critical structures while maintaining effective tumor control. Specifically, we compared the dosimetric outcomes of PAT against IMPT. By addressing the limitations of current proton therapy techniques, this study seeks to explore whether PAT can enhance therapeutic outcomes for esophageal cancer patients while minimizing the risks of radiation‐induced complications.

## 2 METHODS AND MATERIALS

### 2.1 Patient selection

A retrospective cohort of ten patients with histologically confirmed esophageal cancer was selected for this planning study. The cohort encompassed a range of tumor locations, including the upper, middle, and lower thirds of the esophagus. Tumor volumes and nodal involvement varied, reflecting the clinical heterogeneity of esophageal cancer presentations. Detailed patient characteristics, including age, sex, anatomical disease site, and gross target volume, are summarized in Table 1.

**TABLE 1:** Patient demographics and disease characteristics.

PID: Patient ID	Age	Sex	Disease site	Target volume(cc)
1	65	M	Upper third esophagus	51.22
2	62	M	Upper third esophagus & lymph nodes	850.73
3	64	M	Lower third esophagus	260.33
4	77	M	Middle & lower third esophagus	924.56
5	70	M	Lower third esophagus	882.66
6	61	M	Lower third esophagus	474.79
7	86	F	Upper third esophagus & lymph nodes	185.67
8	62	M	Lower third esophagus & lymph nodes	568.15
9	47	M	Lower third esophagus	950.57
10	63	M	Upper third esophagus	147.43

### 2.2 Simulation and target delineation

All patients underwent computed tomography (CT) simulation with intravenous contrast. The scan range extended beyond the entire esophagus based on the location and extent of disease, and images were acquired with a 3 mm slice thickness. Target volumes were delineated in accordance with the consensus guidelines.^23^ Clinical target volumes (CTVs) were determined by the treating radiation oncologist based on tumor location, lymph node involvement, and imaging findings. OARs were contoured and included the lungs, heart, spinal cord, liver, and kidneys.

### 2.3 Treatment planning and evaluation

Two treatment techniques were compared: IMPT and PAT. Both plans were generated using the Monaco treatment planning system (v6.1, Elekta AB, Stockholm, Sweden), incorporating a worst‐case scenario robust optimization approach. Robustness parameters included ± 3.5% range uncertainty and ± 5 mm setup uncertainty. All dose calculations were performed using a Monte Carlo algorithm with a 3 mm dose grid resolution. All dose calculations assumed a constant relative biological effectiveness (RBE) value of 1.1, and no variable or LET‐based RBE modeling was applied in this study. The prescription dose was 50.4 Gy (RBE) delivered in 28 fractions. More specifically, the target dose was planned to achieve 5040 cGy(RBE) covering 95% of the target volume; the OAR constrains included lung: V20 ≤ 30%, V5 ≤65%, lung mean dose ≤ 2000 cGy(RBE), liver mean dose ≤2100 cGy(RBE), heart mean dose ≤3800 cGy(RBE), spinal cord max dose ≤ 4500 cGy(RBE) to 0.03 cc, kidney mean dose ≤ 2000 cGy(RBE). The planning objective was to ensure adequate target coverage while minimizing OAR doses and optimizing the corresponding evaluation metrics. Each plan was prescribed such that at least 95% of the CTV received 100% of the prescription dose. IMPT plans employed single‐field optimization (SFO) using two or three static beams, with beam arrangement tailored to patient anatomy, consistent with standard clinical practice for adult patients undergoing proton therapy. PAT plans were generated using a continuous 360° gantry rotation with a control point spacing of 2°, yielding highly conformal dose distributions through arc‐based delivery.

#### 2.3.1 Dosimetric evaluation

All plans were evaluated with Monaco v6.1 for plan quality. Conformity index (CI) and heterogeneity index (HI) were used to evaluate the dose conformity and homogeneity of the target. CI and HI were defined as follows:

(1) $$CI=\frac{V_{target50.4\;Gy\left(\mathrm{RBE}\right)}}{V_{total50.4\;Gy\left(\mathrm{RBE}\right)}}$$

(2) $$HI=\frac{D_{5}}{D_{95}}$$

Where $V_{target\;50.4\;Gy(\mathrm{RBE})}$ represents target volume covered by 50.4 Gy(RBE), $V_{total50.4\;Gy(\mathrm{RBE})}$ represents the total 50.4 Gy(RBE) volume, D_95_ represents the minimum dose in 95% of the target volume, and D_5_ represents the minimum dose in 5% of the target volume. The closer the CI and HI values are to 1, the better the dose conformity and target dose homogeneity.

Dose metrics were evaluated between two planning groups, such as mean lung dose, percentage of volume over 5 Gy(RBE)(V_5_), and percentage of volume over 20 Gy(RBE)(V_20_) for the lung, mean and max dose for heart, mean dose for liver and kidneys.

#### 2.3.2 Robustness evaluation

Each plan was subjected to 21 worst‐case scenario simulations derived from the defined robustness parameters. For clinical acceptability, at least 90% of the robustness scenarios were required to deliver ≥95% of the prescription dose to ≥95% of the target volume. Additionally, the worst‐case scenario was required to meet a minimum threshold of 90% dose coverage to 90% of the target volume.

#### 2.3.3 LET evaluation

In this study, dose‐average linear energy transfer (LET_d_) was applied to explore the potential clinical benefits of PAT. The Monte Carlo option was chosen to calculate dose‐averaged LET scored to water.^24^, ^25^ The LET_d_ is defined as follows:

(3) $$\;\bar{L_{d_{wat}}}=\frac{\sum_{n=1}^{N}\sum_{s=1}^{S_{n}}\omega_{n}L_{sn,wat}\varepsilon_{sn}{\left(\frac{s}{p}\right)}_{med}^{wat}}{\sum_{n=1}^{N}\sum_{s=1}^{S_{n}}\omega_{n}\varepsilon_{sn}{\left(\frac{s}{p}\right)}_{med}^{wat}}$$

In which N is the total number of particles that deposit energy within the voxel. The index n denotes a single charged particle that traverses the voxel in *Sn* steps. *ω_n_* is the weight of particle n. The index s denotes a specific step in the path of particle n through the voxel. *L_sn_*_,_ and *L_sn_*_,_ *_wat_* are the mean energy lost per unit path length traversed by the particle in medium and water, respectively. *ε_sn_* is electronic energy lost by the particle in step s and ${\left(S/\rho \right)}_{med}^{wat}$ is the mass stopping power of the particle in water divided by the mass stopping power of the particle in the medium.^26^

The average LET_d_ of CTV, lungs, heart, liver and kidneys, maximum LET_d_ of the spinal cord tissue have been evaluated.

#### 2.3.4 Statical analysis

Statistical analyses were performed using SPSS (v24.0, IBM Corp., Armonk, NY, USA). For normally distributed data, paired t‐tests were conducted to compare dosimetric parameters between IMPT and PAT. For non‐normally distributed data, Wilcoxon signed‐rank tests were applied. A two‐sided *p*‐value < 0.05 was considered statistically significant.

## 3 RESULTS

### 3.1 Robustness evaluation

**FIGURE 1:**
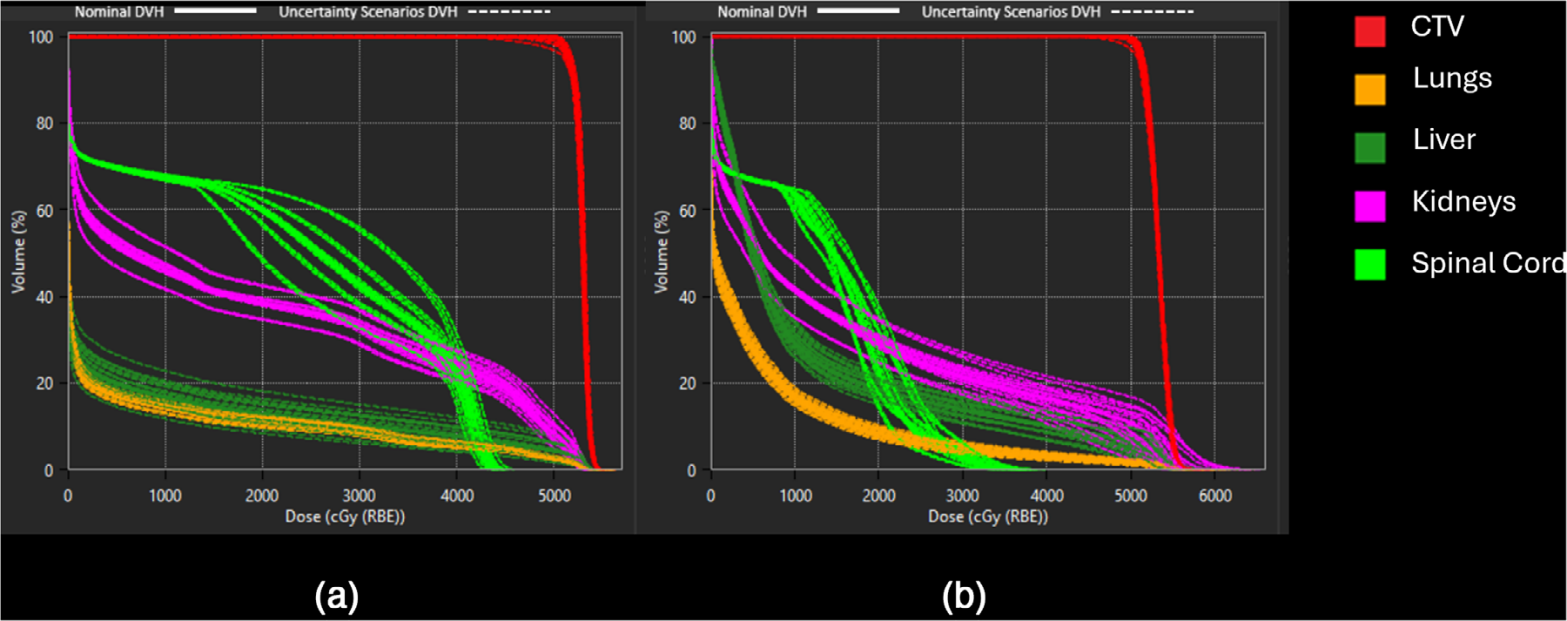
Worst scenarios’ dvh of the target and selected organs‐at‐risk for patient #2. Panel a is the impt plan, and panel b is the proton arc plan.

Robustness evaluations were conducted to ensure adequate target dose coverage under uncertainty. A total of 21 worst‐case scenarios were analyzed, incorporating a ± 3.5% range uncertainty and a 5 mm setup uncertainty, to verify compliance with dose coverage criteria. Figure 1 illustrates the dose‐volume histogram (DVH) perturbations for one representative patient (#2), comparing IMPT and PAT plans under worst‐case conditions.

### 3.2 Nominal plan quality comparisons

**FIGURE 2:**
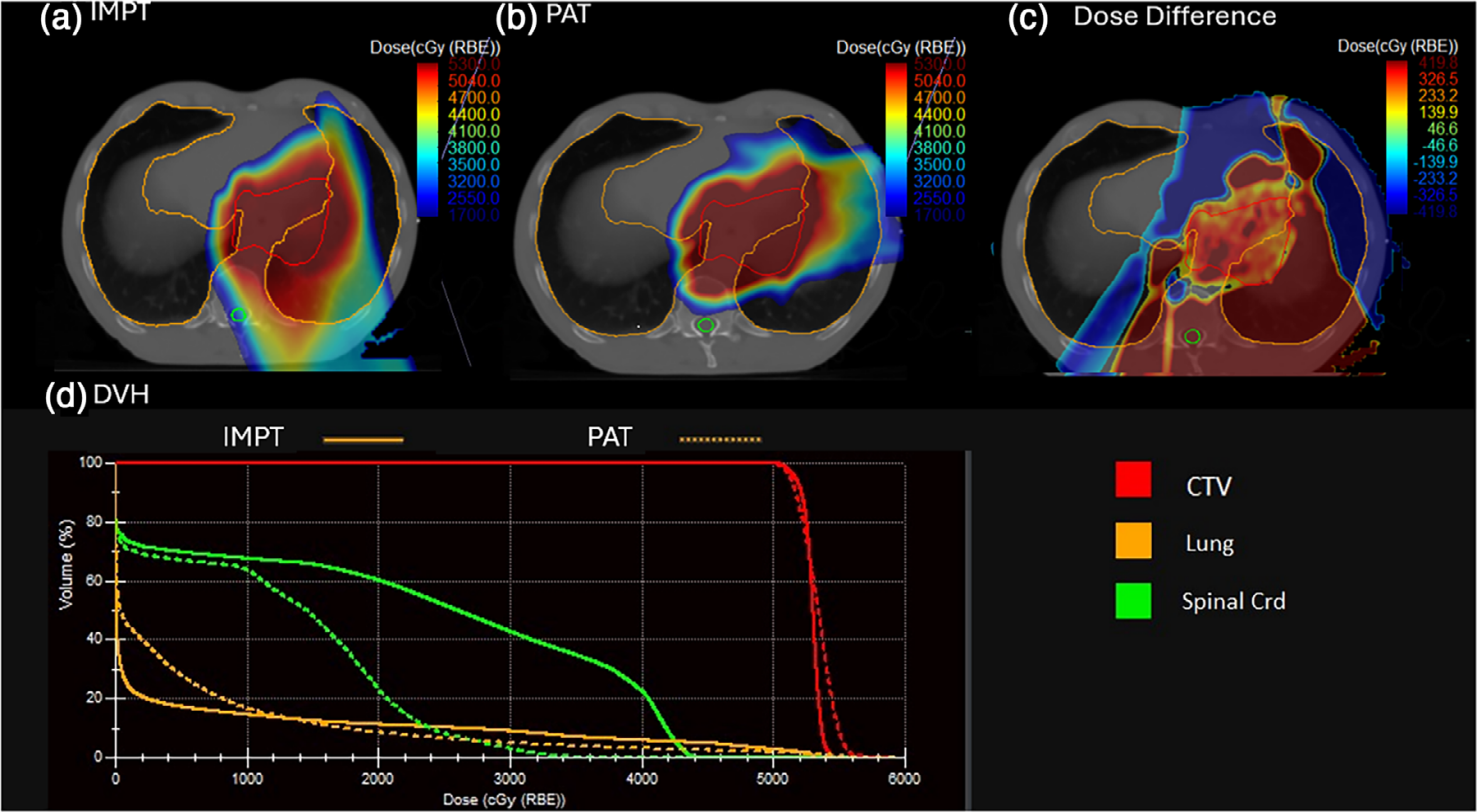
Dosimetric difference and dvh histogram for one example case. a is for impt, b is for pat, c is the dose difference between impt and pat, d is the dvh comparison between impt and pat.

Figure 2 and Table 2 shows the dosimetric differences between IMPT and PAT. Overall, V50.4 Gy(RBE) is comparable between IMPT (98.94 ± 1.57%) and PAT (99.16 ± 0.97), with *p* = 0.36. However, PAT demonstrated significantly improved dose conformity, with the CI increasing from 0.54  ±  0.13 in IMPT to 0.67  ±  0.17 (*p*  <  0.01). Lung dose metrics also favored PAT, with a statistically significant reduction in V20 Gy(RBE) (13.44  ±  10.80% in IMPT vs. 6.88  ±  8.35% in PAT; *p*  <  0.01), indicating potential mitigation of radiation pneumonitis risk. Spinal cord maximum dose was also reduced with PAT(30.93  ±  10.85 Gy(RBE) vs. 23.22  ±  9.55 Gy(RBE); *p*  =  0.02). Other metrics, including mean doses to heart, liver, and kidneys, trended lower with PAT_‐_ but did not reach statistical significance.

**TABLE 2:** Dosimetric and robustness comparison between IMPT and PAT.

		IMPT		PAT		*P* value	
ROI	Evaluation metric	Nominal plan	Robustness (worst‐case scenario)	Nominal plan	Robustness (worst‐case scenario)	Nominal plan comparison (IMPT vs. PAT)	Robustness comparison (worst‐case scenario IMPT vs. PAT)
CTV	V50.4Gy(RBE) (%)	98.94±1.57	95.35%±3.42%	99.16±0.97	94.57%±5.18%	0.36	0.38
	V95	‐	99.21%±1.95%	‐	100.00%±0.00%	‐	0.18
	V90	‐	97.65%±4.08%	‐	99.26%±0.22%	0.18	0.18
	CI	0.54±0.13	‐	0.67±0.17	‐	<0.01	‐
	HI	1.06±0.03	‐	1.05±0.04	‐	0.46	‐
Spinal cord	Max dose (Gy(RBE))	30.93±10.85	36.11±10.21	23.22±9.55	28.19±7.85	0.02	0.07
Lungs	V5 Gy(RBE) (%)	19.51±13.41	22.65%±4.96%	23.62±22.39	32.73%±7.03%	0.21	0.02
	V20 Gy(RBE) (%)	13.44±10.80	13.44%±5.56%	6.88±8.35	8.91%±5.01%	<0.01	0.07
	Mean dose (Gy(RBE))	6.16±4.72	7.75±3.41	5.56±5.75	7.84±4.73	0.07	0.46
Heart	Mean dose (Gy(RBE))	5.56±5.75	7.65±7.26	4.79±4.80	7.05±6.35	0.07	0.25
Liver	Mean dose (Gy(RBE))	2.60±2.69	3.39±3.96	2.64±3.90	4.03±5.12	0.17	0.16
Kidneys	Mean dose (Gy(RBE))	5.31±6.45	8.43±9.52	4.68±5.64	5.22±6.17	0.11	0.09

### 3.3 LET analysis

Table 3 presents the comparison of dose‐averaged LET_d_ between IMPT and PAT plans. Mean LET_d_ within the CTV was slightly higher in PAT(2.61  ±  0.48 keV/µm) compared to IMPT (2.48  ±  0.37 keV/µm), though not statistically significant (*p*  =  0.50). Similarly, maximum LET_d_ to the spinal cord and mean LET_d_ to other OARs did not differ significantly between modalities.

**TABLE 3:** LET_d_ comparison between IMPT and PAT.

ROI	Evaluation metric	IMPT	PAT	*P* value
CTV	Mean(keV/µm)	2.48±0.37	2.61±0.48	0.50
Spinal cord	Max(keV/µm)	4.43±2.41	3.63±1.33	0.37
Lungs	Mean(keV/µm)	1.67±1.21	1.23±0.78	0.34
Heart	Mean(keV/µm)	2.06±1.67	1.21±0.81	0.85
Liver	Mean(keV/µm)	2.21±2.95	1.49±1.48	0.73
Kidneys	Mean(keV/µm)	1.88±1.54	1.72±1.44	0.16

## 4 DISCUSSION

This study explored the clinical potential of PAT for esophageal cancer through a systematic dosimetric comparison with IMPT. The results demonstrate that, relative to IMPT, PAT achieves improved sparing of critical organs, including the heart, lungs, and spinal cord, while maintaining adequate target dose coverage. These results are consistent with previous studies exploring the application of PAT in other cancer types, including prostate and head‐and‐neck cancers.^17^, ^18^

One of the key dosimetric advantages observed in this study is the significant reduction in mean dose to OARs, particularly the lungs and spinal cord, which are critical structures adjacent to the esophageal target. For V5, the PAT is significantly higher than IMPT due to the lower‐dose bath, but V20 is a more significant factor in radiation‐induced pneumonitis,^27^ for which the PAT is significantly lower than IMPT. This outcome suggests that PAT could play a crucial role in reducing the risk of long‐term radiation‐induced complications such as radiation pneumonitis, cardiac toxicity, or esophageal stricture, all of which are common concerns in esophageal cancer treatment​.

Although no statistically significant differences were observed in dose‐averaged LET (LET_d_) between IMPT and PAT plans, a consistent trend was noted in which PAT provided slightly higher LET_d_ within the CTV and reduced LET_d_ to surrounding normal tissues. This observation is consistent with prior studies incorporating LET_d_ into proton arc optimization, demonstrating the potential of proton arc techniques to enhance LET_d_ within the target while reducing LET_d_ exposure to surrounding normal tissues.^28^ These findings suggest that PAT may offer greater control over LET_d_ distribution, potentially allowing more precise shaping of the biological dose. While the clinical implications of these LET_d_ variations remain uncertain, the observed patterns warrant further investigation in larger studies to better understand their potential relevance.

While IMPT has been widely recognized for its precision in proton delivery and tumor conformity, it is associated with limitations related to beam angle restrictions, range uncertainties, and lateral penumbra. These factors can lead to suboptimal OAR sparing and contribute to treatment‐related complications. The introduction of PAT has been proposed as a solution to these challenges by allowing continuous beam delivery from multiple angles. Previous studies have demonstrated dosimetric benefits of PAT over IMPT in prostate cancer, head‐and‐neck cancer, and spine radiosurgery, showing reductions in OAR dose and improved plan robustness.^17^, ^18^, ^19^, ^20^ Recently, Vera et al. reported a PAT planning study in esophageal cancer, demonstrating improved target homogeneity and reductions in key organ‐at‐risk metrics, including the spinal canal, lungs, and heart, compared with IMPT.^22^ They also observed the expected trade‐off of increased low‐dose lung exposure while achieving reductions in intermediate‐ and high‐dose lung metrics, with only minimal changes in integral dose. Importantly, when accounting for setup and range uncertainties as well as breathing motion, the robustness differences between PAT and IMPT were reported to be clinically small, although PAT exhibited modest sensitivity to hotspot metrics in normal tissues. In contrast to that work, the present study evaluates PAT plans generated within Monaco and additionally characterizes LET_d_ distributions, providing complementary evidence of the dosimetric advantages of PAT for esophageal cancer. Our results align with these findings and provide further evidence that PAT offers similar benefits in the treatment of esophageal cancer. Specifically, the continuous gantry rotation in PAT distributes entrance and exit dose over a larger volume, resulting in improved dose conformity to the tumor while reducing concentrated dose deposition to adjacent OARs, in a manner conceptually similar to arc‐based photon technique​. These advantages make PAT a highly promising technique, particularly for anatomically complex regions like the thorax, where multiple critical structures are at risk.

The dosimetric improvements observed with PAT have significant clinical implications. By reducing the radiation dose to OARs, PAT may lower the incidence of acute and late toxicities, allowing patients to better tolerate concurrent chemoradiotherapy and other aggressive treatments. NTCP models^29^ could, in principle, be applied to this cohort to further quantify OAR‐related risk and to estimate potential long‐term clinical impact. However, NTCP modeling for proton therapy remains insufficiently standardized, particularly for esophageal cancer, and existing models are still at an early stage of development and validation.^30^ Therefore, NTCP analysis was not incorporated in the present study. Future work will integrate NTCP modeling once robust, widely accepted proton‐specific models become available, enabling a more comprehensive assessment of long‐term OAR outcomes.

Despite the promising results, there are several limitations to this study. First, the sample size was relatively small, with only ten patients included in the retrospective analysis. A larger cohort would provide more robust evidence of PAT's clinical benefits. Additionally, this study focused solely on dosimetric outcomes; clinical outcomes such as tumor response, survival rates, and patient‐reported quality of life were not assessed. Future prospective clinical trials are needed to validate these findings and assess the long‐term benefits of PAT in esophageal cancer patients.

Another limitation is the reliance on simulation‐based data. Although the robustness of PAT plans was demonstrated through dosimetric comparisons, actual treatment delivery in a clinical setting may introduce additional challenges such as patient positioning, motion management, inter‐fractionation, and equipment limitations. Further research should focus on optimizing these factors to ensure that PAT can be safely and effectively implemented in routine clinical practice.

## 5 CONCLUSIONS

In conclusion, this study highlighted the potential of PAT as a promising technique for improving the treatment of esophageal cancer. PAT offers superior OAR sparing and enhanced dose conformity. These dosimetric advantages may translate into reduced treatment‐related toxicities and better overall patient outcomes. Further research, including clinical trials, is needed to confirm these findings and explore the full clinical potential of PAT for esophageal cancer.

## AUTHOR CONTRIBUTIONS

**Xuanfeng Ding**: Conceptualization; methodology; supervision; review and editing the manuscript. **James Dolan**, **Raymond Dalfson**: Software and planning support. **Martin Soukup**: Software and planning support. **Etienne Lessard**: Software and planning support. **Xiaoda Cong**: Simulation, data acquisition and validation; investigation; writing—original draft; writing—review & editing. **Peilin Liu**: Simulation, data acquisition and validation; investigation; writing—original draft; writing—review & editing. **Ebin Sebastian**: Simulation, data acquisition and validation; investigation; writing—original draft; writing—review & editing. **Peter Y. Chen** Review and edit the manuscript; provide clinical guidance and suggestions. **Rohan L. Deraniyagala**: Review and edit the manuscript; provide clinical guidance and suggestions. **Xiaoqiang Li**: Review and edit the manuscript; provide clinical guidance and suggestions. **Craig W. Stevens**: Review and edit the manuscript; provide clinical guidance and suggestions.

## CONFLICT OF INTEREST STATEMENT

Xuanfeng Ding received honorariums from IBA and Elekta's speaker bureau outside of work presented here. Xuanfeng Ding received industrial research funding from IBA and Elekta. Xuanfeng Ding and Xiaoqiang Li have a patent related to particle arc therapy and was assigned to Corewell Health. There is a license agreement with IBA.

## Supporting information




**Supporting Information**: acm270607‐sup‐0001‐Table_S01.docx
