# Supplementary material for: Advancing proton therapy: Dosimetric superiority of proton arc therapy over IMPT in esophageal cancer treatment
Source: J Appl Clin Med Phys. 2026 May 5;27(5):e70607. doi: 10.1002/acm2.70607 (PMC13143515; doi:10.1002/acm2.70607)
Supplement: Supplementary file 1 — Supporting Information: acm270607‐sup‐0001‐Table_S01.docx [file ACM2-27-e70607-s001.docx]

Table s1. NTCP evaluation for heart, lungs and kidneys

|  |  | IMPT | PAT |
| --- | --- | --- | --- |
|  | End point | NTCP(%) | NTCP(%) |
| Heart | Grade 2 Pericardium | 0.00%±0.00% | 0.00%±0.00% |
| Lungs | Grade 2 radiation pneumonitis | 1.67%±1.33% | 1.91%±2.38% |
| Kidneys | Grade 2 CKD | 32.68%±14.17% | 9.55%±8.63% |
